# Supplementary material for: IL-21-mediated reversal of NK cell exhaustion facilitates anti-tumour immunity in MHC class I-deficient tumours
Source: Nat Commun. 2017 Jun 6;8:15776. doi: 10.1038/ncomms15776 (PMC5467212; doi:10.1038/ncomms15776)
Supplement: Supplementary Information — Supplementary Figures and Supplementary Table [file ncomms15776-s1.pdf]

Supplementary Figures

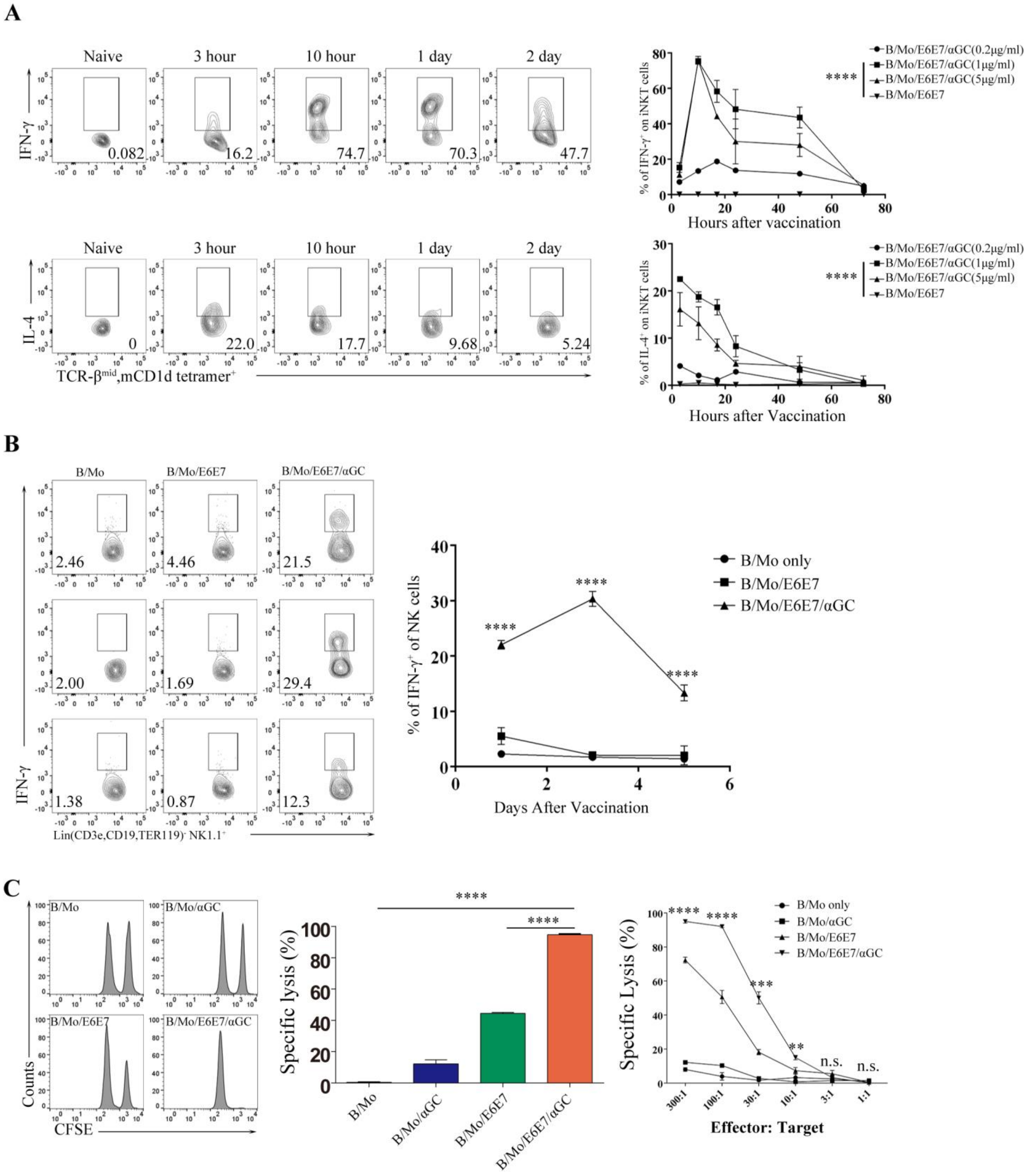

Supplementary Figure 1. NKT ligand-loaded tumour antigen-presenting B cell- and monocyte-based vaccine induces NKT, NK and CD8 T cell responses. (A) The cytokine profiles of liver iNKT cells were assessed in B/Mo/Adk35-E6E7/αGC-injected mice at various times. The levels of IL-4 and IFN-γ were detected by flow cytometry. (**B)** Twelve days after TC-1 WT cell implantation, IFN-γ production by tumour-infiltrating NK cells was assessed in B/Mo-, B/Mo/Adk35-E6E7- and B/Mo/Adk35-E6E7/αGC-injected mice. (**C)** C57BL/6 mice were vaccinated with the indicated form of B/Mo. One week later, in vivo CTL assays were performed by injecting CFSE-labelled syngeneic targets. $CFSE^{high}$, peptide pulsed target; $CFSE^{low}$, peptide unpulsed control. In vitro assays were performed using vaccinated mice splenocytes that were restimulated with the HPV16 E6/E7 peptide mixture (E6: EVYDAFRDL, E7: RAHYNIVTF), and cytotoxicity against TC-1 cells was measured using a standard ^{51}Cr release assay. The data shown are from at least 2 individual experiments with similar results. The data in A, B and C were analysed by two-way ANOVA with Bonferroni multiple comparison tests. *P<0.05, **P<0.01, ***P<0.001, ****P<0.0001.

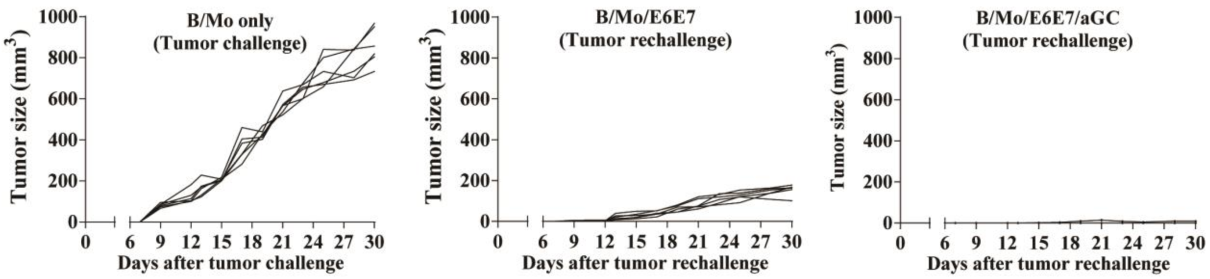

Supplementary Figure 2. Memory antitumour responses induced by vaccination. (A) Tumour-free mice from Fig. 1A and naïve mice (n=6) were vaccinated with the indicated cellular vaccine at day 6 after TC-1 tumour s.c. rechallenge ($1x10^5$) on the opposite flank as on day 0. The data shown are from at least 2 individual experiments with similar results.

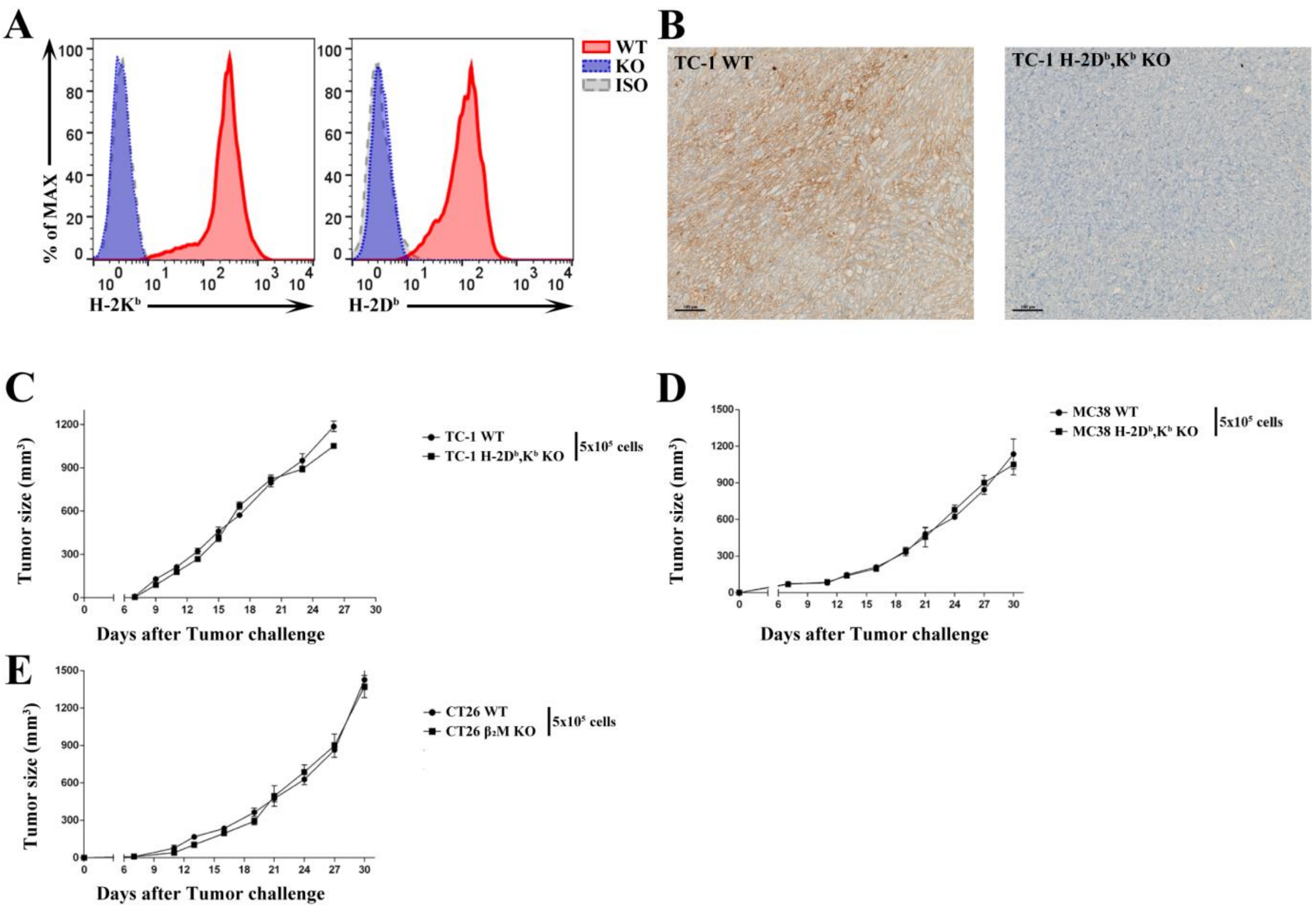

Supplementary Figure 3. H-2D^{b}, K^{b} or Beta 2 microglobulin knock out tumour cells. (A) The expression levels of H-2D^{b} and H-2K^{b} on TC-1 WT or TC-1 H-2K^{b},D^{b} KO cells were measured by flow cytometry. **(B)** The H-2D^{b} expression level in TC-1 WT or TC-1 H-2K^{b},D^{b} KO-bearing mouse tumour (n=5) sections was detected by immunohistochemistry (H2-D^{b}: brown, tumour: blue, Scale bar: 100μm). **(C-E)** The tumour size was measured after s.c. implantation of the indicated tumour cells. The data shown are from at least 2 individual experiments with similar results.

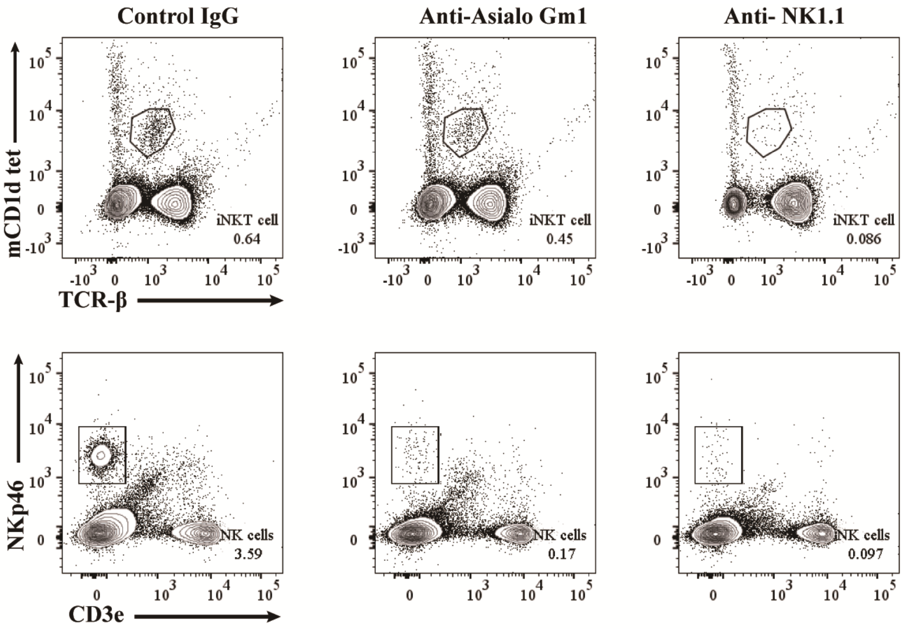

Supplementary Figure 4. Efficacy of NK, iNKT cell depletion by anti-Asialo-GM-1 or anti NK1.1 (PK136). The tumour bearing mice were treated with control IgG, anti-NK1.1 (PK136) or anti-asialo GM-1 to deplete NK cells on day 12 after tumour injection (TC-1). NK cells and NKT cells were detected in the spleen by flow cytometry.

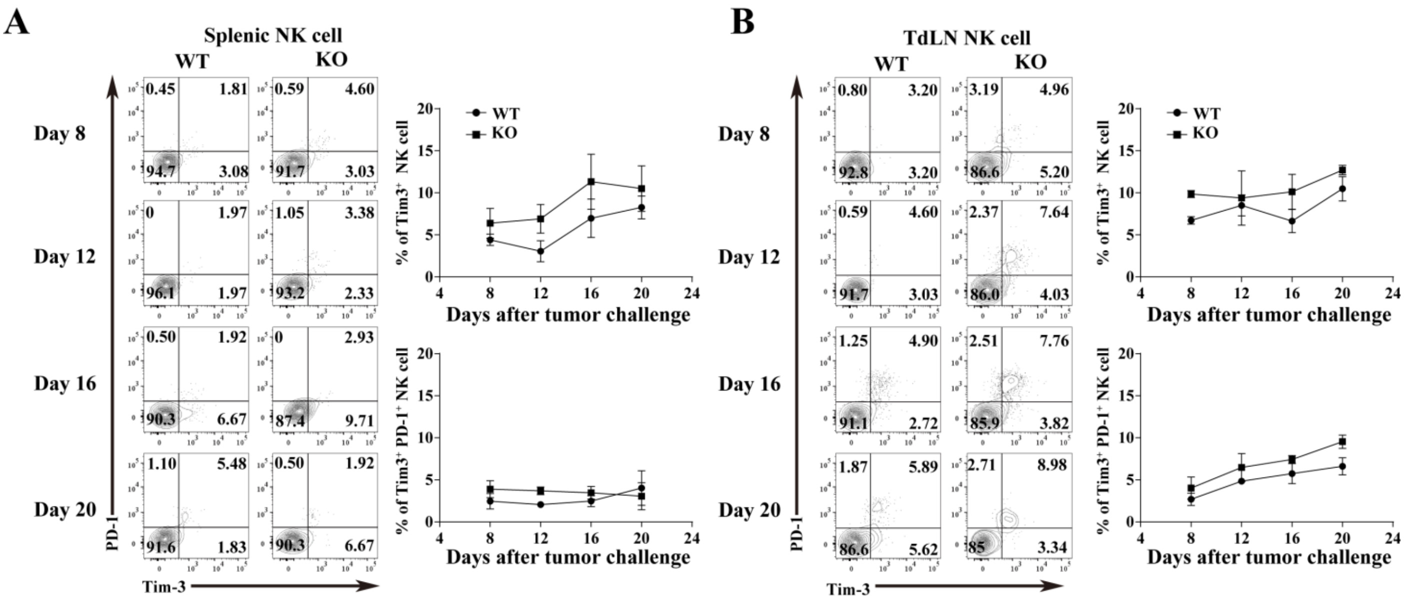

Supplementary Figure 5. Tim-3 and PD-1 expression on natural killer cells in the indicated organs. The kinetic expression levels of Tim-3 and PD-1 on natural killer cells from TC-1 WT or H-2K^b,D^b KO tumour-bearing mice (n=5) were analysed by flow cytometry.

A

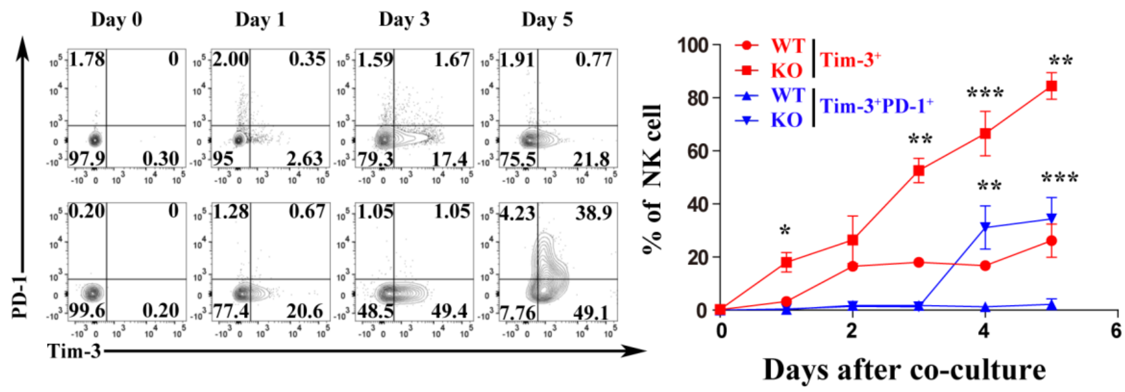

B

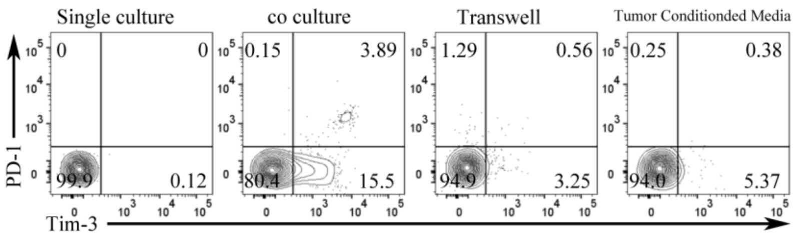

Supplementary Figure 6. Tim-3 and PD-1 expression on NK cells in vitro. (A) The daily kinetic Tim-3 and PD-1 expression levels on NK cells were analysed after MC38 WT or H-2K^b,D^b KO tumour cells were cocultured in vitro with splenic NK cells from C57BL/6 mice. **(B)** The expression levels of Tim-3 and PD-1 on NK cells were assessed after coculture with TC-1 H-2K^b,D^b KO cells via transwell or the addition of tumour-conditioned medium. The data were analysed using a two-tailed unpaired Student's t test *P<0.05, **P<0.01, ***P<0.001.

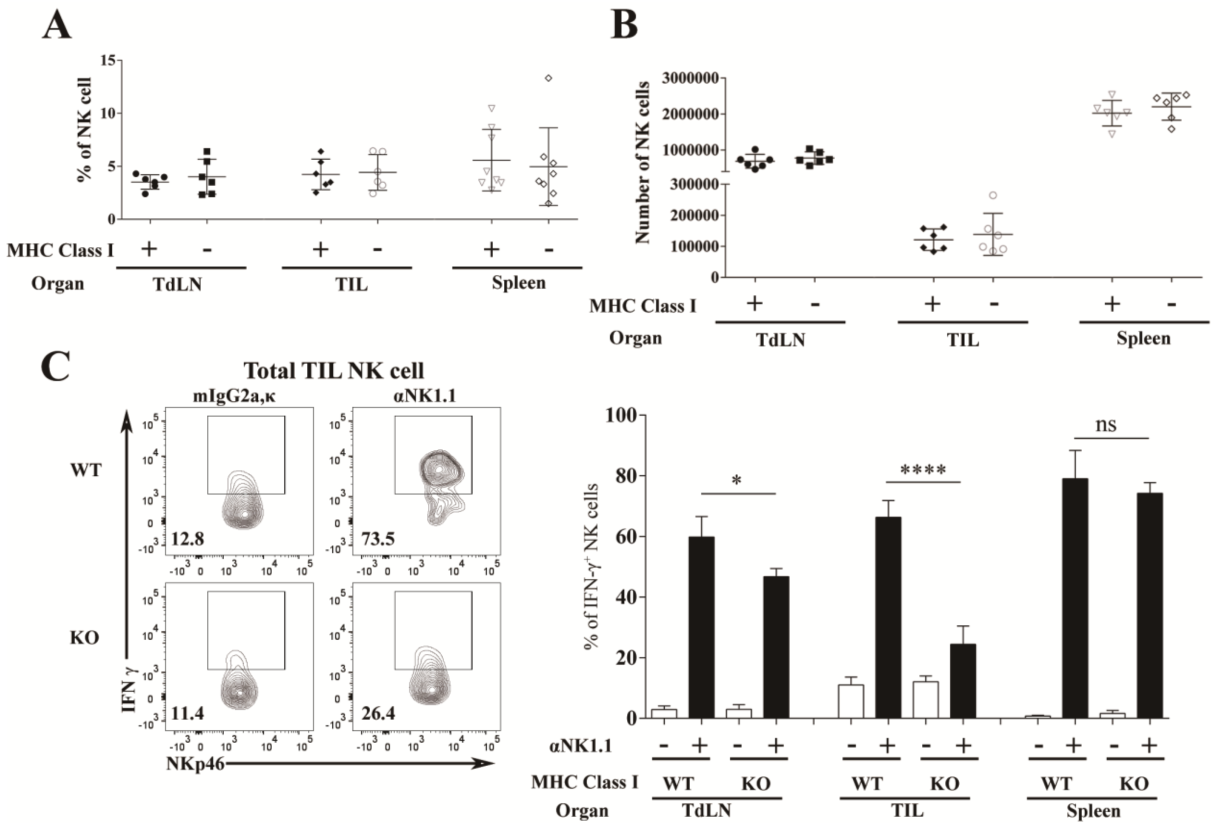

Supplementary Figure 7. Percent, number and function of NK cells in MHC class I-deficient tumours. (A-C) Twelve days after TC-1 WT or H-2K^b,D^b KO cell implantation, **(A)** the percent and **(B)** number of NK cells were analysed in the indicated organs. **(C)** Lymphocytes from the indicated organs were restimulated in vitro with anti-NK1.1 or control IgG for 30 minutes, and IFN-γ production was then evaluated by flow cytometry. The data shown are from at least 2 individual experiments with similar results. The data in C were analysed using a two-tailed unpaired Student's t test. *P<0.05, **P<0.01, ***P<0.001, ****P<0.0001.

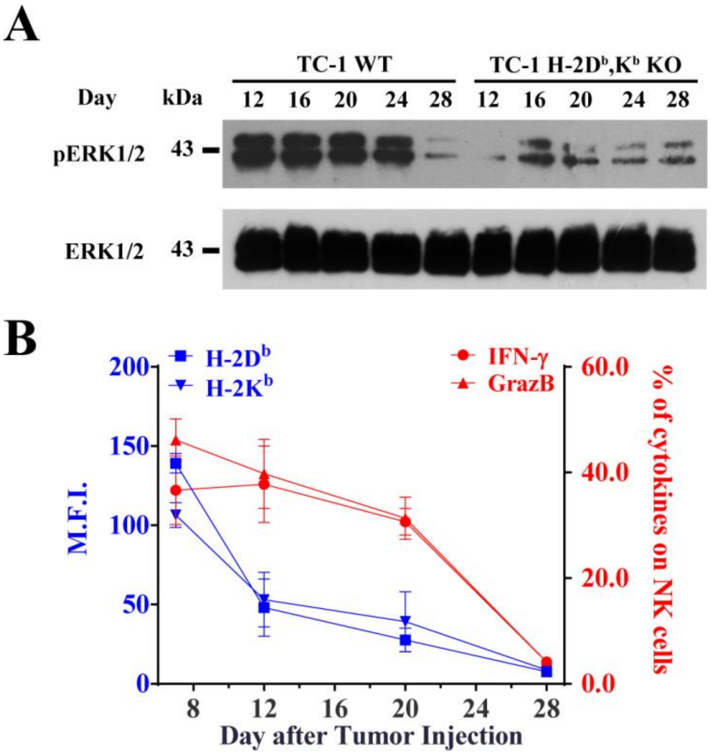

Supplementary Figure 8. ERK activation kinetics and functional analysis of TC-1 WT or H-2K^b,D^b tumour-infiltrating NK cells. (A) ERK1/2 phosphorylation was detected by western blot analysis after restimulation in vitro with anti-NK1.1 or control IgG antibody for 30 minutes with TC-1 WT or H-2K^b,D^b KO implantation (5x10^5)-infiltrating NK cells at different time points (B) The expression of H-2K^b, D^b , IFN-γ and Granzyme B on tumour cells or intratumoural NK cells from mice bearing TC-1 WT tumours was measured over time.

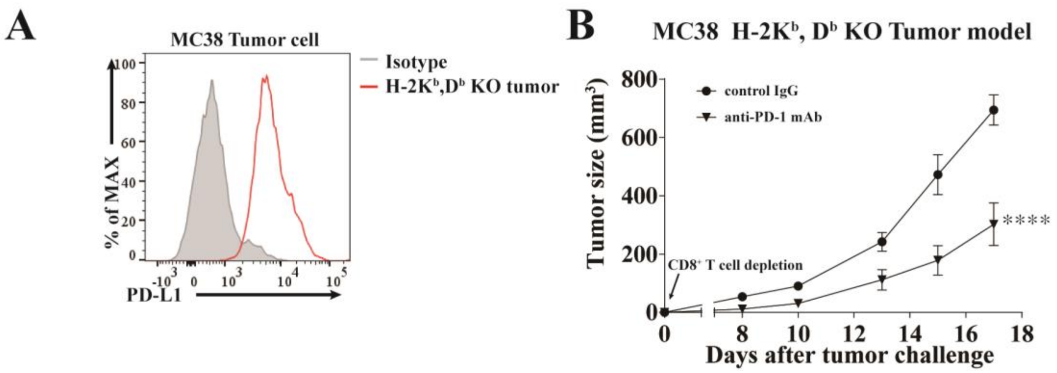

Supplementary Figure 9. The PD-1/PDL1 axis transmits a negative signal on exhausted NK cells.

(A) The expression level of PDL-1on MC38 H-2K^b, D^b KO was measured by flow cytometry. **(B)** MC38 H-2K^b, D^b KO tumour cells (1x10^5) were implanted into mice, which were then treated with anti-PD-1 (300 µg) or control IgG twice weekly. A depleting antibody (2.43) for CD8+ T cell depletion was injected i.p. twice weekly. The data shown are from at least 2 individual experiments with similar results. The data in B was analysed by two-way ANOVA with Bonferroni multiple comparison tests. *P<0.05, **P<0.01, ***P<0.001, ****P<0.0001.

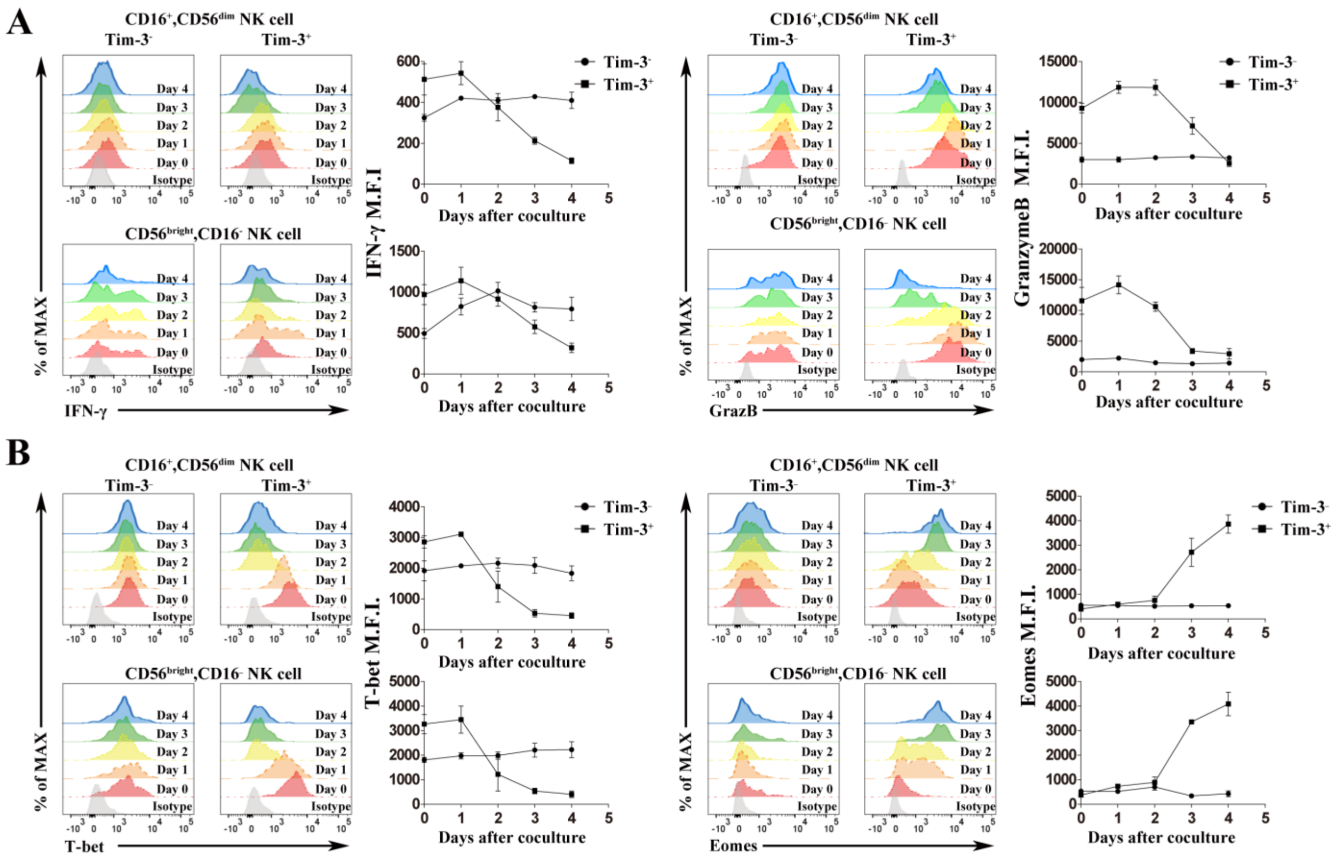

Supplementary Figure 10. HeLa β2m KO cells induce dysfunction of human NK cells. (A-B) Tim-3+ or Tim-3- human NK cells cocultured with HeLa β2m KO cells for 3 days were restimulated in vitro with human ULBP-2, and IFN-γ and granzyme B production and T-bet and Eomes expression were assessed by flow cytometry. The data shown are from at least 2 individual experiments with similar results.

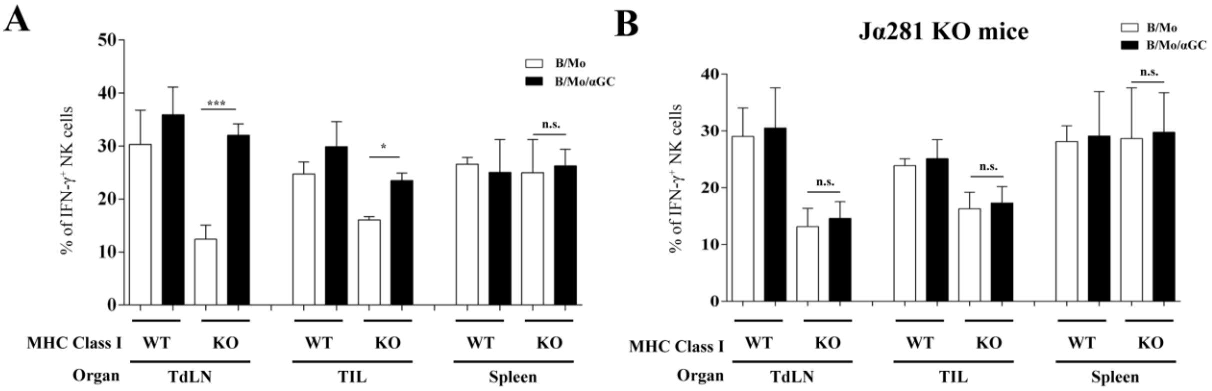

Supplementary Figure 11. Exhausted NK cells induced in MHC class I knockout tumour-bearing mice are reversed by NKT-dependent activation. (A-B) Eleven days after TC-1 WT or H-2K^b,D^b KO cell implantation, each tumour-bearing **(A)** C57BL/6 or **(B)** Jα281 KO mouse was vaccinated with B/Mo or B/Mo/αGC (1x10^6). In addition, 12 hours later, lymphocytes from the indicated organs were stimulated with control IgG or anti-NK1.1 for 30 minutes, and IFN-γ production by NK cells was analysed by flow cytometry. The data were analysed using a two-tailed unpaired Student's t test. *P<0.05, **P<0.01, ***P<0.001, ****P<0.0001.

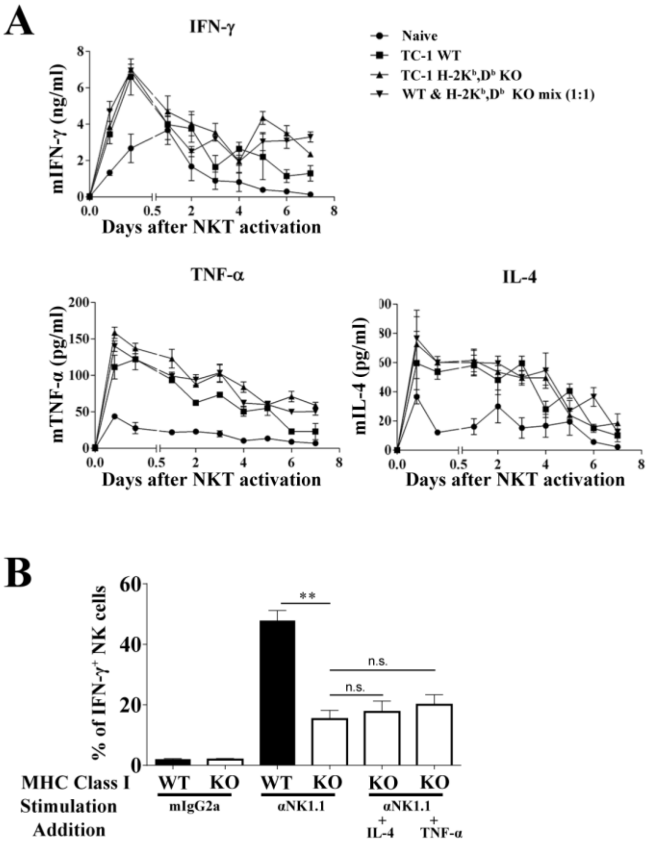

Supplementary Figure 12. NKT cells induce various cytokines in tumour-bearing mice. (A) The cytokine profile in the serum of B/Mo/αGC-injected WT, TC-1WT, TC-1 H2-$K^b D^b$ KO or WT&H-$2K^b$,D^b KO mixed tumour-bearing mice at various times. The levels of IFN-γ, TNF-α and IL-4 were detected by ELISA. **(B)** Twelve days after TC-1 WT or H-$2K^b$,D^b KO implantation, intratumoural lymphocytes were stimulated in vitro with TNF-α (20 ng/ml) and IL-4 (20 ng/ml) and then restimulated with anti-NK1.1. The data were analysed using a two-tailed unpaired Student's t test. *P<0.05, **P<0.01, ***P<0.001, ****P<0.0001.

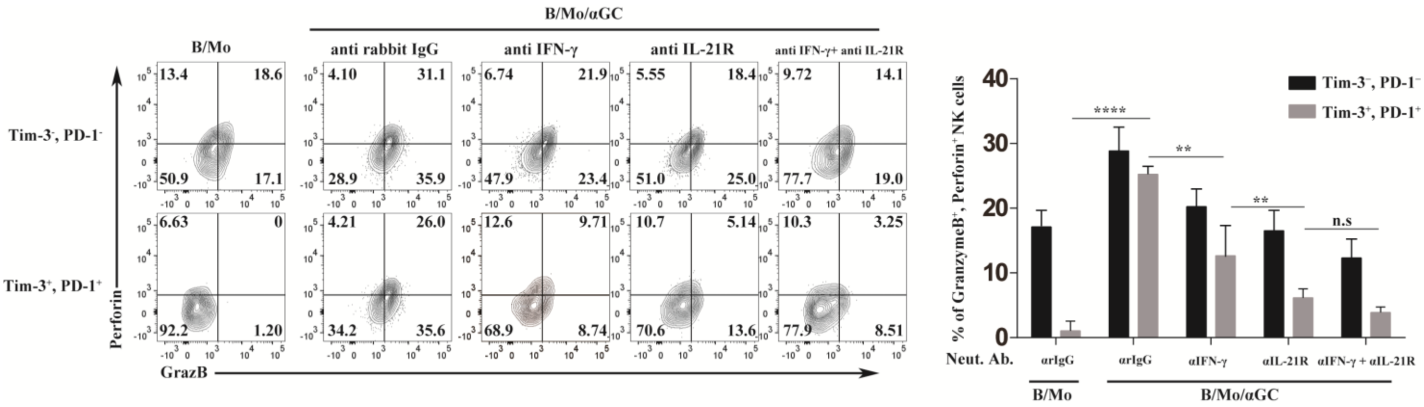

Supplementary Figure 13. IL-21 drives the functional reversal of Tim-3^{+}PD-1^{+} NK cells. Nine days after TC-1 H-2K^{b},D^{b} KO implantation, anti-IFN-γ (500 μg/mouse) or anti-IL-21R (300 μg/mouse) or both were injected via i.p., and two days later B/Mo/αGC (1x10^{6} cells/mouse) was injected i.v. with anti-IFN-γ (500 μg/mouse) or anti-IL-21R (300 μg/mouse) or both. In addition, after an additional 12 hours, infiltrating lymphocytes were restimulated in vitro with anti-NK1.1, and the production of perforin and granzyme B was assessed by flow cytometry. The data were analysed using a two-tailed unpaired Student's t test. *P<0.05, **P<0.01, ***P<0.001, ****P<0.0001

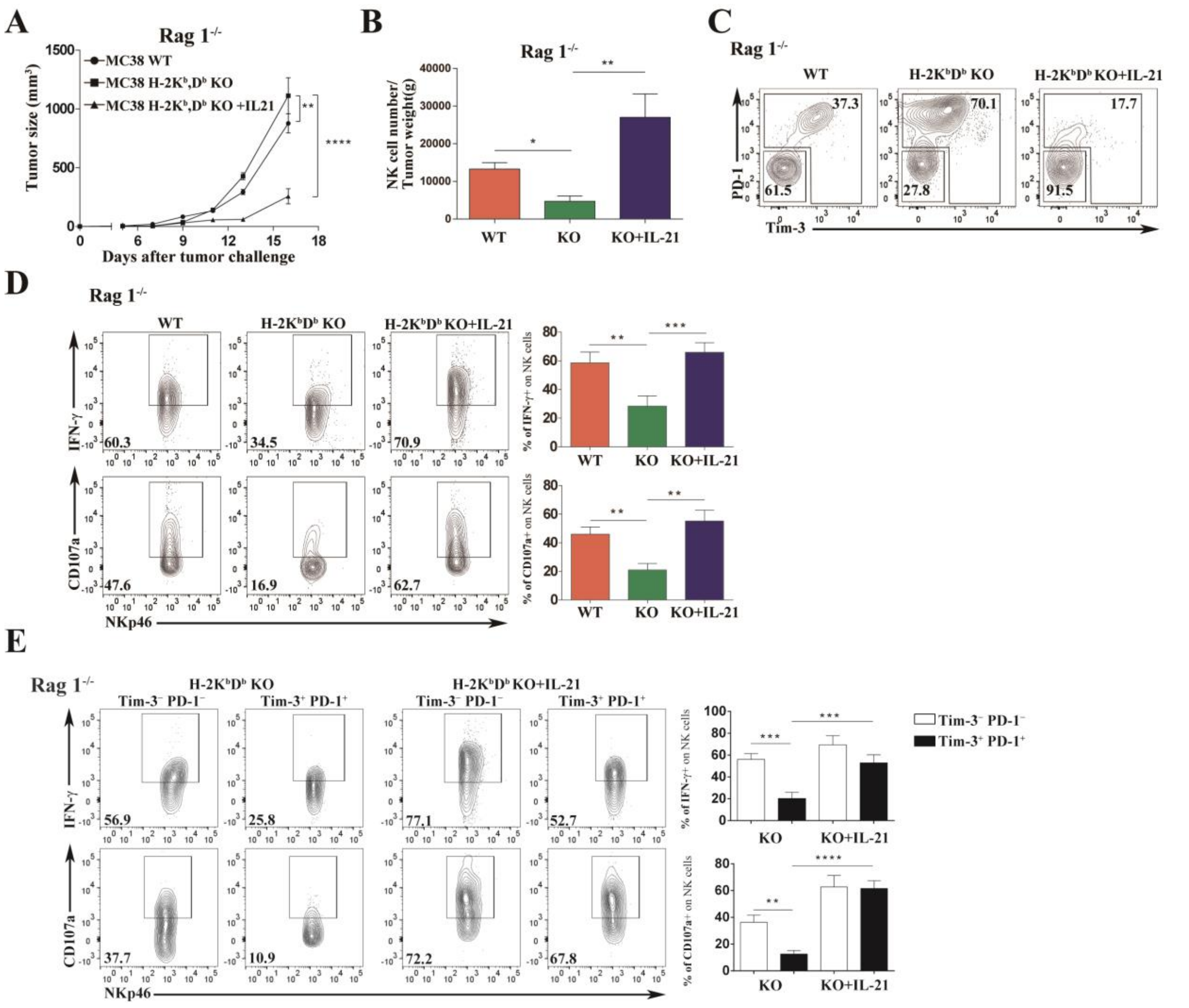

Supplementary Figure 14. Intratumoural injection of IL-21 induces functional reversal of exhausted Tim-3$^+$ PD-1$^+$ NK cells in Rag$^{-/-}$ mice. (A-E) MC38 WT- or H-2K^b,D^b KO-bearing Rag 1$^{-/-}$ mice (n=5) were treated with IL-21 (10 μg/mouse) by intratumoural injection every 3 days from days 9 to 15. **(A)** Tumour growth was measured using a metric calliper 2-3 times per week. (**B**) The number of NK cells was analysed as ($= \frac{\%\ \text{of CD45.2 NKp46 cells x No.of tumor infiltrating lymphocyte}}{Tumor\ weight\ (g)}$). **(C-E)** Tumour-infiltrating lymphocytes were restimulated using anti-NK1.1, and **(C)** the expression of Tim-3 and PD-1 and **(D-E)** the production of IFN-γ and CD107a were analysed in the indicated cell populations. The data were analysed using a two-tailed unpaired Student's t test. *P<0.05, **P<0.01, ***P<0.001.

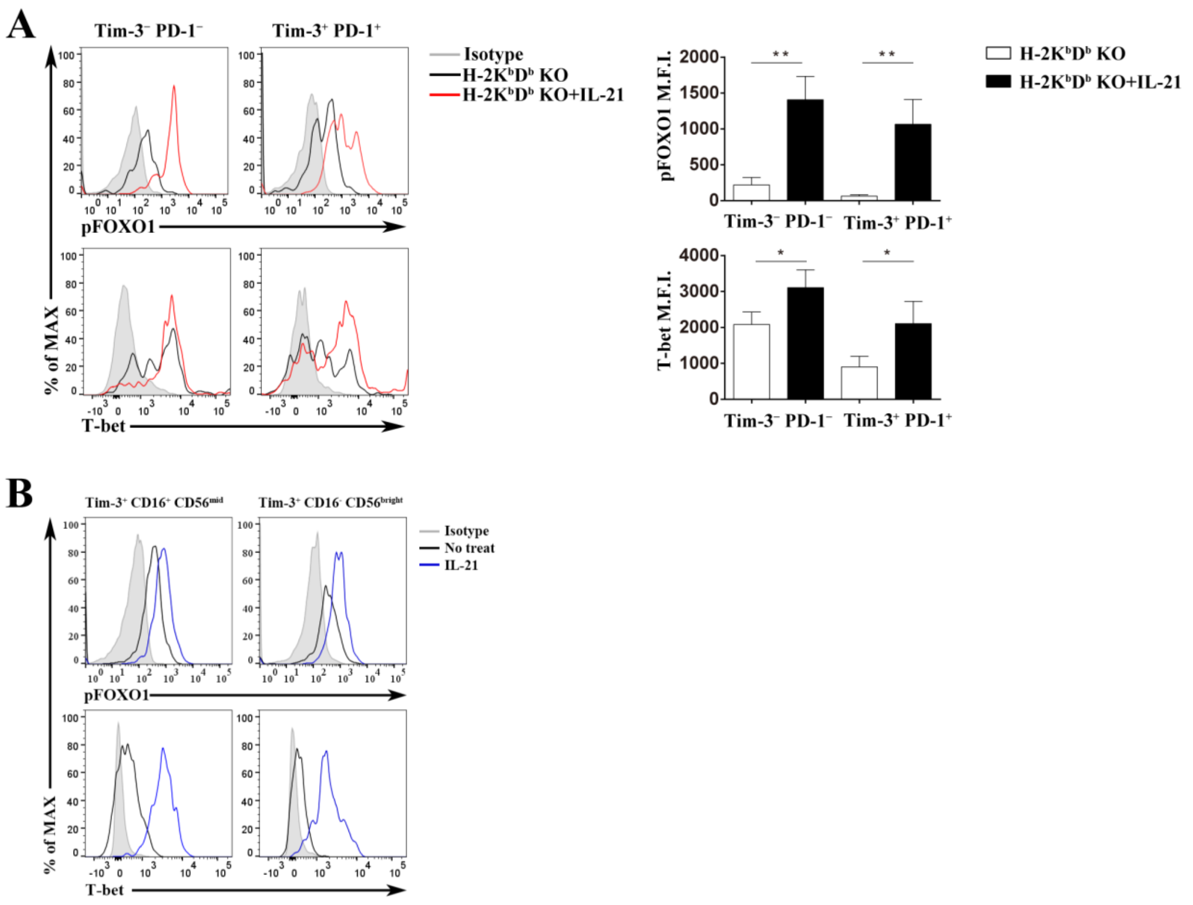

Supplementary Figure 15. IL-21 elicits Foxo1 phosphorylation and T-bet upregulation in mice and humans. (A) Intracellular staining of pFOXO1 and T-bet in Tim-3$^+$PD-1$^+$ and Tim-3$^-$PD-1$^-$ NK cells from MC38 H-2K^b,D^b KO-bearing Rag 1$^{-/-}$ mice that were treated with IL-21 (10 μg/mouse) by intratumoural injection every 3 days. (B) Intracellular staining of pFOXO1 and T-bet in the indicated NK cells stimulated with IL-21 (50 ng/ml) in vitro for 1 hour. The data were analysed using a two-tailed unpaired Student's t test. *P<0.05, **P<0.01, ***P<0.001.

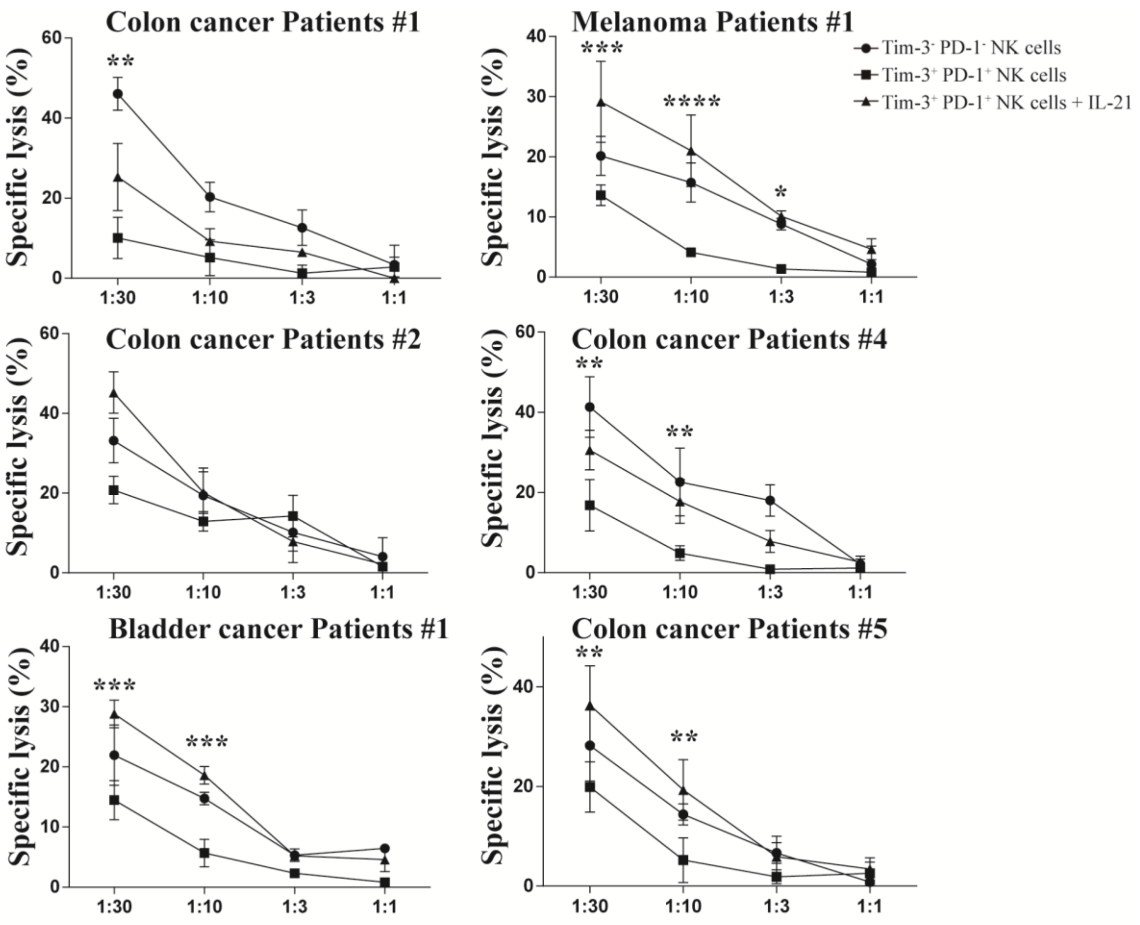

Supplementary Figure 16. *In vitro* cytotoxicity of intratumoural NK cells from cancer patients. Isolated Tim-3$^+$PD-1$^+$ or Tim-3$^-$PD-1$^-$ NK cells were incubated overnight in the presence or absence of rIL-21 (20 ng/ml) and cocultured with ^{51}Cr-labeled K562 cells as target cells. The data were analysed by two-way ANOVA with Bonferroni multiple comparisons tests. *P<0.05, **P<0.01, ***P<0.001, ****P<0.0001

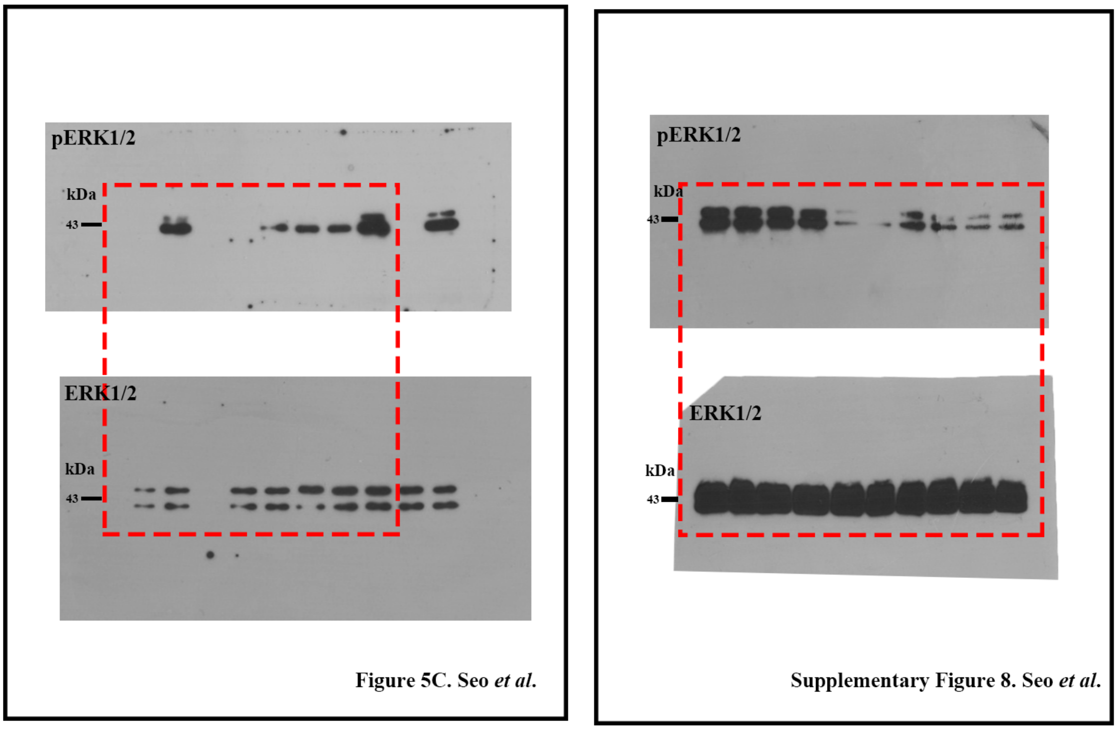

Supplementary Figure 17. Original western blot image for Figure 5C and Supplementary Figure 8A.

Antibody	Clone	Dilution	Manufacturer	Cat#
H-2Db	KH95	1:100	Biolegend	1115
H-2Kb	AF6-88.5	1:100	Biolegend	1165
PD-1	RMP1-14	1:100	Biolegend	1141
PDL-1	10F.9G2	1:100	Biolegend	1243
Tim-3	RMT3-23	1:50	Biolegend	1197
CD3ε	145-2C11	1:100	Biolegend	1003
CD19	6D5	1:100	Biolegend	1155
Gr-1	RB6-8c5	1:100	Biolegend	1084
TER-119	TER-119	1:100	Biolegend	1162
NKp46	29A1.4	1:50	Biolegend	1376
NK1.1	PK136	1:50	Biolegend	1087
CD45.2	104	1:100	Biolegend	1098
CD45	2D1	1:100	Biolegend	3685
CD45	HI30	1:100	Biolegend	3040
CD11b	M1/70	1:100	Biolegend	1012
CD27	LG.3A10	1:100	Biolegend	1242
Ly49D	4E5	1:100	Biolegend	1383
CD226	10E5	1:100	Biolegend	1288
CD69	H1.2F3	1:100	Biolegend	1045
CD150	A12	1:100	Biolegend	3063
TIGIT	1G9	1:100	Biolegend	1421
CD226	11A8	1:100	Biolegend	3383
2B4	m2b4(B6)45	1:100	Biolegend	1335
CD49b	DX5	1:100	Biolegend	1089
EpCam	9C4	1:100	Biolegend	3242
p44/42 MAPK (Erk1/2)	137F5	1:1000	Cell Signaling	4695
Phospho-p44/42 MAPK (Erk1/2) (Thr202/Tyr204)	197G2	1:1000	Cell Signaling	4377
Phospho-FoxO1 (Ser256)		1:100	Cell Signaling	9461

Antibody	Clone	Dilution	Manufacturer	Cat#
NKG2A/C/E	20d5	1:100	ebioscience	5896
PD-1	J43	1:100	ebioscience	9985
KLRG1	2F1	1:100	ebioscience	5893
2B4	eBio244F4	1:100	ebioscience	2441
IFN-γ	XMG1.2	1:50	ebioscience	7311
CD107a	eBio1D4B	1:100	ebioscience	1071
Granzyme B	NGZB	1:50	ebioscience	8898
Perforin	eBioOMAK	1:50	ebioscience	9392
T-bet	eBio4B10	1:100	ebioscience	5825
Ly49A/D	12A8	1:100	ebioscience	5783
Eomes	Dan11mag	1:100	ebioscience	4875
NKG2D	1D11	1:100	ebioscience	5878
CD69	FN50	1:100	ebioscience	0699
CD160	BY55	1:100	ebioscience	1609
HLA-ABC	W6/32	1:100	ebioscience	9983
PD-1	MIH4	1:100	ebioscience	9969
Tim-3	F38-2E2	1:100	ebioscience	3109
TIGIT	MBSA43	1:100	ebioscience	9500
KLRG1	13F12F2	1:100	ebioscience	9488
IFN-γ	4S.B3	1:50	ebioscience	7319
Granzyme B	GB11	1:50	ebioscience	8899
Eomes	WD1928	1:100	ebioscience	4877
Fixable Viablility Dye		1:1000	ebioscience	65-0865
pAKT(pT308)	J1-223.371	1:50	BD bioscience	558275
pSTAT1(pY701)	4a	1:50	BD bioscience	562069
pSTAT3(pS727)	4/P-STAT3	1:50	BD bioscience	562072
pERK1/2(pT202/pY204)	20A	1:50	BD bioscience	561992

Supplementary Table 1. Antibody information for flow cytometry and western blot.
